# Exploration of the Solubility Hyperspace of Selected Active Pharmaceutical Ingredients in Choline- and Betaine-Based Deep Eutectic Solvents: Machine Learning Modeling and Experimental Validation

**DOI:** 10.3390/molecules29204894

**Published:** 2024-10-16

**Authors:** Piotr Cysewski, Tomasz Jeliński, Maciej Przybyłek

**Affiliations:** Department of Physical Chemistry, Pharmacy Faculty, Collegium Medicum of Bydgoszcz, Nicolaus Copernicus University in Toruń, Kurpińskiego 5, 85-096 Bydgoszcz, Poland; tomasz.jelinski@cm.umk.pl (T.J.); m.przybylek@cm.umk.pl (M.P.)

**Keywords:** solubility, green solvents, DES, machine learning, COSMO-RS, σ-profiles, sulfonamides

## Abstract

Deep eutectic solvents (DESs) are popular green media used for various industrial, pharmaceutical, and biomedical applications. However, the possible compositions of eutectic systems are so numerous that it is impossible to study all of them experimentally. To remedy this limitation, the solubility landscape of selected active pharmaceutical ingredients (APIs) in choline chloride- and betaine-based deep eutectic solvents was explored using theoretical models based on machine learning. The available solubility data for the selected APIs, comprising a total of 8014 data points, were collected for the available neat solvents, binary solvent mixtures, and DESs. This set was augmented with new measurements for the popular sulfa drugs in dry DESs. The descriptors used in the machine learning protocol were obtained from the σ-profiles of the considered molecules computed within the COSMO-RS framework. A combination of six sets of descriptors and 36 regressors were tested. Taking into account both accuracy and generalization, it was concluded that the best regressor is nuSVR regressor-based predictive models trained using the relative intermolecular interactions and a twelve-step averaged simplification of the relative σ-profiles.

## 1. Introduction

Active pharmaceutical ingredients (APIs) are the biologically active components of drugs that produce the intended therapeutic effects. They are the key compounds responsible for diagnosing, treating, or preventing diseases in patients [1,2]. The development and optimization of APIs are central to pharmaceutical research, as their chemical properties, such as stability, solubility, and bioavailability, significantly influence a drug’s safety, efficacy, and dosage. Solubility is indeed one of the critical parameters in the development and characterization of active pharmaceutical ingredients [3,4,5], which affects the full lifecycle of a drug, starting with its synthesis and ending with its administration to the patient [6,7,8]. For an API to exert its pharmacological effect, it must first dissolve in bodily fluids, enabling its absorption into the bloodstream and subsequent distribution to target sites. Poor solubility can limit a drug’s absorption, reducing its bioavailability [9,10] and potentially necessitating higher doses, which can lead to adverse effects or therapeutic failure [11,12,13]. Consequently, optimizing solubility is a fundamental aspect of drug formulation and delivery, ensuring that the APIs achieve the desired clinical outcomes while maintaining safety and efficacy profiles [14,15,16,17]. Following this line of thought, many techniques have been proposed to enhance the solubility of poorly dissolvable APIs [18,19,20,21]. While experimental measurements are necessary in order to obtain a full understanding of the behavior of a particular API in a selected solvent, the prediction of solubility is also extremely useful, particularly during the development of a drug [22,23,24,25,26]. If the number of potentially useful solvents can be narrowed down by an initial predictive and screening stage, it is particularly valuable from an environmental perspective since it reduces the chemicals and energy needed for additional experiments. This approach fits perfectly with the concept of “green chemistry” [27,28], and it is no surprise that it was widely adopted [29,30,31]. The need for accurate, reliable, and relatively fast prediction methods focused the attention of researchers on neural networks and machine learning [32,33]. These techniques have already found numerous applications in the pharmaceutical domain, including solubility predictions [34,35,36,37].

The “green chemistry” approach mentioned above establishes not only the reduction of the environmental impact by limiting the number of laboratory operations, but also the introduction of more environmentally friendly solvents, the so-called “green solvents” [38,39]. These solvents, characterized by a lower toxicity and enhanced biodegradability, have quickly found widespread usage and become an alternative to the classical organic solvents [40,41]. Among the different solvents that offer environmentally safety, the deep eutectic solvents (DESs) are one of the most proficient dissolution media [42,43,44,45]. These systems can be broadly defined as mixtures of two or more components, usually containing a hydrogen bond acceptor (HBA) and a hydrogen bond donor (HBD), with the melting point of the mixture being lower than the melting points of the individual constituents [46,47]. DESs combine a high solubilizing potential for many APIs with desirable properties, which includes a high potential for tuning [48,49,50,51,52]. The pharmaceutical industry has embraced the beneficial effects of deep eutectic solvents, which have found numerous applications in this field [53,54,55,56,57,58,59,60,61].

The current study is focused on the development of an effective and reliable predictive model for solubility estimation of active pharmaceutical ingredients in deep eutectic solvents based on choline chloride and betaine. For this purpose, the available solubility data space of 15 selected APIs was explored and supplemented with new experimental data. The COSMO-RS approach was employed to obtain a set of descriptors that characterize the system under consideration and were used in the machine learning protocol. This procedure resulted in the obtainment of the predictive model used for the exploration of the solubility hyperspace of the selected APIs.

## 2. Results and Discussion

### 2.1. Experimental Extension of the Solubility Dataset in DESs

The solubility dataset of various active pharmaceutical ingredients in different DES formulations is quite extended. Our research group has contributed to these efforts by measuring the DES solubility of such APIs as ibuprofen and ketoprofen [62], ferulic acid [63], curcumin [64], caffeine [65], theobromine [66], theophylline [67], dapsone [68], edaravone [69], as well as various sulfonamides, including probenecid, sulfamethazine, sulfamethoxazole, sulfasalazine, sulfacetamide, and sulfanilamide [70]. The undisputed advantage of this particular dataset is its consistency, both in terms of the applied measurement protocol and the used eutectic formulations. Nonetheless, there is always room for extending the known solubility space of APIs by conducting new measurements, and such an extension, comprising 128 new data points, is provided here.

**Figure 1:**
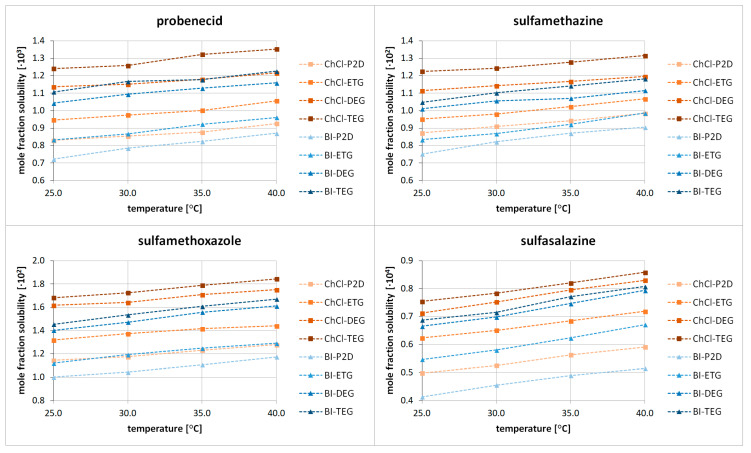
Mole fraction solubilities of four different sulfonamides in DESs composed of choline chloride (ChCl) or betaine (BI) with 1,2-propanediol (P2D), ethylene glycol (ETG), diethylene glycol (DEG), or triethylene glycol (TEG) in a 1:2 molar ratio at various temperatures.

New solubility values were obtained for four sulfonamides, namely probenecid (PC), sulfamethazine (SMZ), sulfamethoxazole (SMA), and sulfasalazine (SSZ). Eight distinct eutectic compositions were used by combing two hydrogen bond acceptors (HBAs), i.e., choline chloride (ChCl) and betaine (BI) with four hydrogen bond donors (HBDs), namely 1,2-propanediol (P2D), ethylene glycol (ETG), diethylene glycol (DEG), and triethylene glycol (TEG). The HBA:HBD molar ratio was set to 1:2, and the solubilities were measured at four different temperatures, namely 25 °C, 30 °C, 35 °C, and 40 °C. The obtained solubility values are collected in Figure 1 and in Table S1 in the Supplementary Materials.

**Figure 2:**
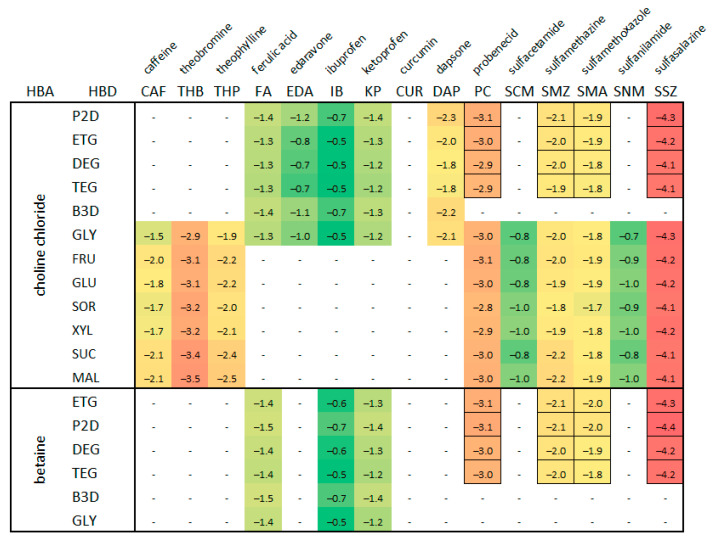
Collection of the API solubility values, expressed as the decadal logarithm of the mole fraction, in choline chloride and betaine deep eutectic solvents in ambient conditions. Newly measured values are marked with black borders. Only the 1:2 proportion of HBA and HBD was included. Colors map the span of solubility values. APIs include the following: caffeine (CAF), theobromine (THB), theophylline (THP), ferulic acid (FA), edaravone (EDA), ibuprofen (IB), ketoprofen (KP), curcumin (CUR), dapsone (DAP), probenecid (PC), sulfacetamide (SCM), sulfamethazine (SMZ), sulfamethoxazole (SMA), sulfanilamide (SNM), and sulfasalazine (SSZ). HBDs: 1,2-propanediol (P2D), ethylene glycol (ETG), diethylene glycol (DEG), triethylene glycol (TEG), 1,3-butanediol (B3D), glycerol (GLY), fructose (FRU), glucose (GLU), sorbitol (SOR), xylitol (XYL), sucrose (SUC), and maltose (MAL).

The general picture emerging from the obtained results shows that sulfamethoxazole is characterized by the highest solubility in the studied eutectic formulations, followed closely by sulfamethazine. The solubility of probenecid is about one order of magnitude lower, while the solubility of sulfamethoxazole has been found to be the lowest among the studied sulfonamides. This general observation is in accordance with the solubilities of these compounds in the DES systems studied earlier (see Figure 2). When it comes to the efficiency of the studied eutectic systems, it can be concluded that eutectics utilizing choline chloride have a better overall performance than those with betaine. The influence of the type of HBD is also very important from the perspective of sulfonamide solubility. In all studied cases, triethylene glycol proved to be the most efficient, followed by diethylene glycol, ethylene glycol, and 1,2-propanediol. When considering all the possible systems, a general decreasing trend of solubility can be seen, namely the following: ChCl-TEG > ChCl-DEG > BI-TEG > BI-DEG > ChCl-ETG > ChCl-P2D > BI-ETG > BI-P2D. Also, quite obviously, the temperature increase promotes the solubility of all the sulfonamides. The mole fraction solubilities at 25 °C for the best-performing ChCl-TEG system were the following: x_SMA_ = 0.01681, x_SMZ_ = 0.01225, x_PC_ = 0.00124, and x_SSZ_ = 0.000075. The detailed results can be found in the Supplementary Materials.

The above results should be placed in the context of the solubility values previously obtained for various APIs. Such a comparison is provided in Figure 2, which shows the collection of solubilities of the considered solutes in different DES formulations. For clarity of presentation, only the results obtained for the 1:2 molar proportion of HBA:HBD are shown, and the temperature is restricted to 25 °C.

The coloring scheme allowed a direct comparison of the solubilities at room temperature. For example, SSZ has the lowest solubility among all the studied solids and augmenting the pool of data by adding the results corresponding to the new HBDs does not change this this observation. Similarly, the solubility of SMZ and SMA measured for the purpose of this project also are comparable with the previously published studies. Additionally, Figure 2 documents the significant differences in the absolute solubilities among the included APIs. This shows that DES formulations can often improve the solubility of solutes relative to other solvents, but at the same, they time can be restricted by the generally low solubility of a particular API. Despite this, deep eutectic solvents can be regarded as both universal and effective solvent systems, which has been documented numerous times. For example, in the case of caffeine [65], the optimal DES composition achieved a solubility equal to 165% of the best-performing organic solvent, i.e., DMSO. Similarly, dimethyl sulfoxide, which is the most effective classical solvent for many APIs, was outperformed by theophylline and theobromine [66,67]. In the case of dapsone [68] and edaravone [69], the best organic solvents, i.e., acetone and dichloromethane, respectively, were also inferior to many DES compositions. Of course, the results are not always that impressive, as it was evidenced in the case of COX inhibitors [62]. While for ibuprofen DESs indeed outperformed the best-performing chloroform, for ketoprofen, rather surprisingly, they could not compete with methanol. Of course, one has to keep in mind that many solvents, although offering a high dissolution potential for many solutes, are not pharmaceutically acceptable. This emphasizes the advantages of DESs, which can be applied in the pharmaceutical industry, even if they offer a slightly smaller solubilizing efficiency. Also worth mentioning is the often dramatic increase in API solubility when comparing DES to water. The most striking example is curcumin [64], for which the most efficient eutectic was characterized by a 12-thousand-times higher solubility when compared to an aqueous solution. The environmental and health safety impact, although sometimes disputed to some extent [71,72,73,74], is also an important factor that favors eutectics. Of course, the difference in performance observed among the deep eutectic solvents is related to their composition. The results obtained for various APIs in different eutectic systems point out some general trends. It seems that such hydrogen bond donors as glycerol, triethylene glycol, and diethylene glycol can be regarded as particularly efficient and worth consideration in future studies. Also, in terms of hydrogen bond acceptors, choline chloride performed generally better than betaine. The above considerations clearly show the importance of DESs as dissolution media, as well as their potential for tuning to specific applications.

### 2.2. COSMO-RS Derived Solubility

It is commonly accepted [75,76,77] that the COSMO-RS (Conductor-like Screening Model for Realistic Solvation) is a reliable tool for thermodynamic properties characteristics of bulk systems. Although formally this approach is restricted to fluid systems, it can be extended to treating the solid liquid equilibria (SLE) if the experimental or estimated values of fusion data are provided. This makes the COSMO-RS very attractive for solubility computations. Unfortunately, one encounters problems with this approach, seriously limiting its applicability even for qualitative solubility guesses. There are two major obstacles, originating both from the inaccuracy of the model and also from experimental limitations. A key experimental limitation inherent to all models based on the thermodynamic principles of the dissolution process is the inability to determine the fusion properties for compounds that degrade before reaching their melting point, sublime, or undergo polymorphic or pseudopolymorphic transformations [65,78,79,80,81,82]. Additionally, the COSMO-RS is incapable of predicting the solubility of certain APIs in many cases, for example benzenesulfonamide [83], ferulic acid [63], or caffeine [65]. In such cases, a high concentration of the solutes in the saturated solutions prevents them from using the fast iterative method as it fails to converge, incorrectly predicting the total miscibility. The application of the alternative approach relying on the solution of the full SLE problem is quite time-consuming, and even this type of computation fails occasionally since non-physical immiscible liquid–liquid phases are predicted. Fortunately, this is not a common situation for typical organic solvents although it is encountered for the saturated systems in DESs. Hence an alternative way to determine solubility such as the application of machine learning-based models employing the COSMO-RS descriptors becomes attractive [63,83].

**Figure 3:**
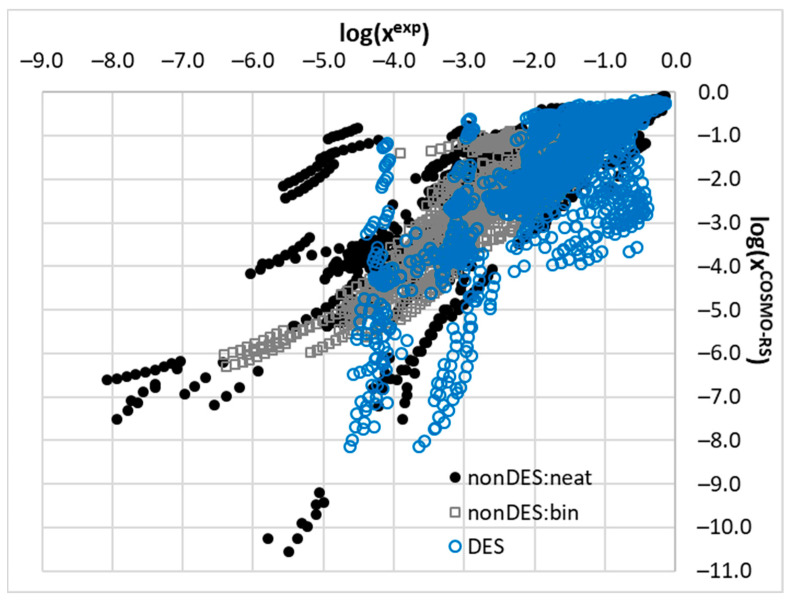
Relationship of experimental solubility and values computed using COSMO-RS for all the included APIs in neat solvents, binary solvent mixtures, and all studied DES systems. The temperature relationships and concentration dependencies of saturated solutions were taken into account.

To support the above statements, a graphical representation of the relationships between the computed and measured solubility values is provided in Figure 3. It is evident that the COSMO-RS is unable to predict solubility in many cases as the majority of the computed values suffer from serious inaccuracies especially in the DESs. Among the three studied subsets, the values for the solubility of the binary mixtures were computed with the highest accuracy, which might be attributed to the quite limited range of solvents used for the measurements. This is quite understandable, since a majority of the solvent pair combinations might lead to the immiscibility of the solvents, preventing solubility measurements.

**Figure 4:**
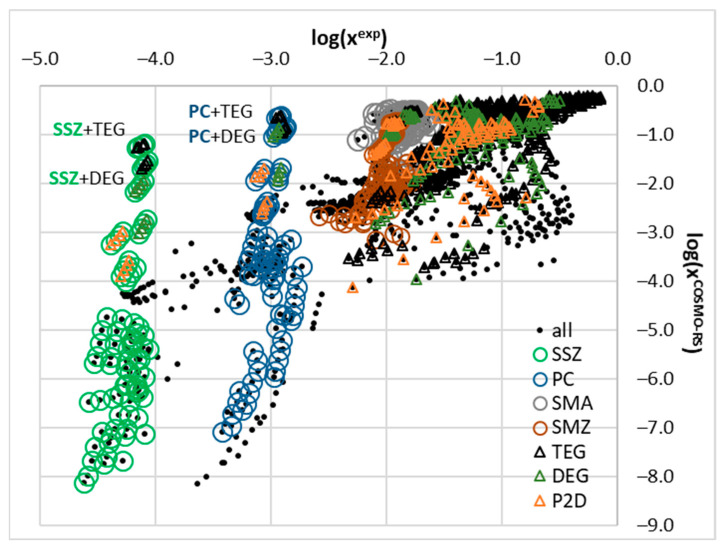
Relationship of experimental solubility and values computed using COSMO-RS for the subset of data presented in Figure 1, characterizing only APIs in DES systems. Open circles mark newly measured sulfonamides, and open triangles point out selected HBD counterparts of the studied DESs. For acronyms and their meanings, refer to Figure 1.

Since the main focus of this paper is on DESs, a closer inspection of this particular subset might be interesting. In Figure 4, the computed and measured solubility data are plotted for pointing out some additional limitations of COSMO-RS applicability as the source of the estimated solubility data. The plots were overlaid with open circles and open triangles as markers of the data corresponding to the new sulfonamides’ measurements and the most efficient HBD, respectively. It is quite surprising that the solubility computed for sulfasalazine (SSZ) and probenecid (PC) are very inaccurate. It happened that these two compounds belong to a class of aromatic carboxylic acids and as such might interact with alcohols and polyalcohols such as DEG, and TEG. Interestingly, ibuprofen, ketoprofen, and ferulic acid also possess a carboxylic group but the deviation between the computed and measured values is significantly less prominent. This seems to be an interesting aspect worth subsequent study for further experimental and theoretical considerations.

Being aware of the above-mentioned limitations of the COSMO-RS-derived solubility, the authors could not use such values as a direct source of the physical characteristics of the saturated systems as it is incapable of directing a proper selection of new solvents for screening purposes. However, the computed values often can be treated as a reliable source for the physicochemical characteristics of the saturated systems that enables the inclusion of the computed values, such as the valuable descriptor, for machine learning. The significant contribution of such computed solubility values to the model was already documented in our earlier works [62,63].

### 2.3. Machine Learning Solubility Model

The primary motivation for utilizing machine learning is to develop a robust model that can reliably estimate the solubility for systems not yet studied experimentally. This model is to be used as a guide for further experimental measurements and to reduce investigational efforts. By focusing on the most promising systems with a high likelihood of success, new measurements can be directed efficiently. This approach might include exploring not only different combinations of active pharmaceutical ingredients, hydrogen bond acceptors, and hydrogen bond donors but also varying proportions of deep eutectic solvent constituents and temperature dependencies. Expanding the dataset used for model development is therefore crucial for capturing the relevant factors that influence solute–solvent interactions.

In our previous studies, we examined several combinations of two different HBAs with a variety of HBDs. This led to the optimization of many practically useful solvents that meet the general requirements for pharmaceutical applications as effective dissolution media. However, despite considerable scientific effort, exploring the vast solvent hyperspace remains a challenging task, as only a small fraction of the potential combinations have been investigated. Figure 2 illustrates this limitation, showing that we covered less than 50% of the possible systems while using only a single ratio of HBA:HBD = 1:2. Clearly, exploring other solvent component ratios, even when restricting the investigation to the specified HBAs, HBDs, and water, significantly reduces the known region, leaving much more to be explored if necessary.

Hence, an extensive tuning of 36 regressor hyperparameters was conducted for each of the six descriptor sets to identify the models that not only best fit the experimental solubility data but also exhibited the highest predictive capability. In addition to the standard regularization parameters inherent to many regression models, a custom scoring function was employed. This function assessed the training subset’s accuracy using few key metrics: the mean absolute error (MAE), as well as a penalty for the number of outliers and the number of formally not acceptable values as mentioned in the methodology Section 3.6. The final model’s accuracy was evaluated using a test subset, consisting of 20% of the solubility data that were not included in the training process.

After an extensive non-linear models search, the process of regressor selection was undertaken for excluding those models that poorly represented the experimental solubility data. This resulted in the elimination of the most regressors, leaving only a few for further evaluation. The second selection criterion was an assessment of their overfitting and generalization capabilities, which was determined by comparing the accuracies of the test subsets. The models that exhibited a low mean absolute error on the training subset, but a high error on the test subset were deemed overfitted and were subsequently excluded. This group included the models generated using for example the MLPRegressor neural network, CatBoost, gradient boosting, and the XGBoost regressors. Following this analysis, the best model, demonstrating both high accuracy and strong predictive performance, was identified as the nu-Support Vector Regressor (nuSVR), a machine learning algorithm often used for regression tasks. This approach is based on the principles of Support Vector Machines (SVMs) and allows for the control of both the number of support vectors and the margin of tolerance through a parameter called “nu”, which ranges from zero to one. This flexibility makes the nuSVR effective for modeling complex, non-linear relationships, as it can balance model complexity and accuracy [84]. Recently, the nuSVR was applied successfully, predicting the solubility of aqueous solutions [85] and organic solvents [86].

**Figure 5:**
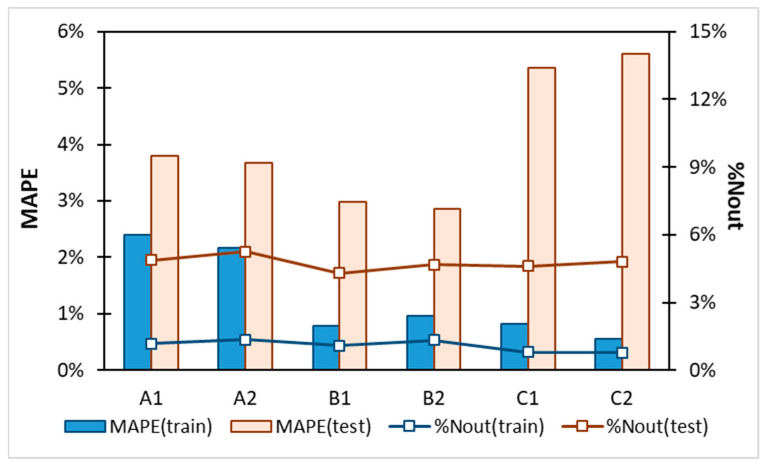
Comparison of nuSVR model’s performance, the parameters of which were tuned on all six descriptors sets. Lines represent the percentage of outliers and bars stand for MAPE of train and test subsets.

**Figure 6:**
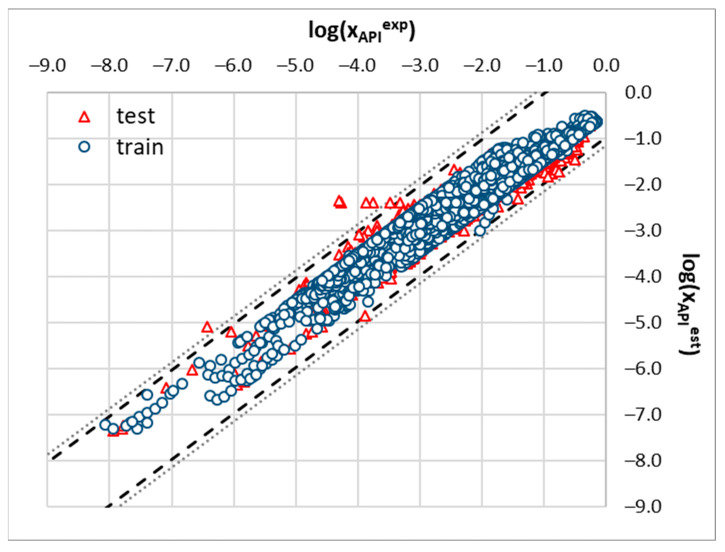
Correlation between experimental solubility values (N = 8014) and those computed using the NuSVR regressor trained on the B2 set of descriptors. Dotted and dashed lines represent values corresponding to three times the standard deviation computed for the whole dataset or test subset, respectively.

Figure 5 and Figure 6 present the final results of the nuSVR model. Instead of the mean absolute error, the mean absolute percentage error was used, as it provides a more intuitive measure of accuracy. A key observation from Figure 5 is that the choice of descriptor set has a significantly greater impact on the overall accuracy than the inclusion of the solubility data computed using the COSMO-RS. This finding is promising as it suggests that the model can be effectively applied to cases where fusion data are unavailable, thereby reducing the preparation time by omitting the solubility calculations. As illustrated in Figure 3 and Figure 4, the computed solubility values for many of the saturated systems in this study exhibit significant inaccuracies, which does not help in model training. While the COSMO-RS has demonstrated satisfactory accuracy in previous studies, including those by our group, this is not generally the case for deep eutectic solvents. Thus, omitting the solubility values from the descriptor set is recommended based on our findings.

Another noteworthy conclusion drawn from Figure 5 is that the full σ-potential representation performed the worst among the tested cases. Conversely, the simplest representation using a six-step function failed to capture the essential information, making it suboptimal. The twelve-step averaging approach, which provides a balanced solute–solvent description while minimizing overfitting, is the most effective. Therefore, the descriptor set denoted as B2 is identified as the most efficient and accurate for the non-linear model training of the APIs’ dissolution in DES and non-DES solvents.

## 3. Materials and Methods

### 3.1. Materials

Four sulfonamides were used to extend the solubility dataset of the APIs in the DESs. These were as follows: probenecid (PC, CAS: 57-66-9, MW = 285.36 g/mol), sulfamethazine (SMZ, CAS: 57-68-1, MW = 278.33 g/mol), sulfamethoxazole (SMA, CAS: 723-46-6, MW = 253.28 g/mol), and sulfasalazine (SSZ, CAS: 599-79-1, MW = 398.39 g/mol). The deep eutectic solvent formulations involved in this study consisted of a hydrogen bond acceptor (HBA) and a hydrogen bond donor (HBD). Two different HBAs were used, namely choline chloride (ChCl, CAS: 67-48-1) and betaine (BI, CAS: 107-43-7). As for the HBDs, they were represented by four polyols, i.e., ethylene glycol (ETG, CAS: 107-21-1), diethylene glycol (DEG, CAS: 111-46-6), triethylene glycol (TEG, CAS: 112-27-6), and 1,2-propanediol (P2D, CAS: 57-55-6). The above compounds, both the solutes and DES constituents, were supplied by Sigma Aldrich (Saint Louis, MO, USA) and had a purity of ≥99%. Additionally, methanol was used as a supplementary solvent, which was delivered by Avantor Performance Materials (Gliwice, Poland) with a purity of ≥99%. The supplied chemicals were used without any initial procedures, apart from the choline chloride, which was dried before use.

### 3.2. Solubility Determination

The solubility measurements were preceded by the determination of the calibration curves for the studied sulfonamides. Stock solutions of these compounds were prepared in methanol and subsequently diluted. The obtained series of solutions with decreasing concentrations were then measured spectrophotometrically using an A360 spectrophotometer from AOE Instruments (Shanghai, China) in the wavelength range of 200 nm to 500 nm. Their characteristic wavelengths were determined, and the corresponding absorbance values were plotted against the concentrations of the solutions. Three separate curves were prepared, and the obtained values were averaged. The calibration curves were validated in terms of their linearity, expressed by the determination coefficient R^2^, as well as the limits of detection (LOD) and quantification (LOQ). Table 1 shows the details of the calibration curves, and based on the obtained validation values, it can be concluded that all the curves are characterized by a satisfactory linearity, as well as detection and quantification limits below the actual concentrations in the studied samples.

For determining the solubility of the studied sulfonamides in different eutectic compositions, a standard shake-flask procedure was applied [66,87,88,89].

The first step in this procedure involved the preparation of the deep eutectic solvents, which was performed by combining a hydrogen bond donor with a hydrogen bond acceptor. The HBA:HBD molar ratio was set to 1:2. The two HBDs, namely ChCl and BI, together with the four HBAs, namely TEG, DEG, ETG, and P2D, resulted in a total of 8 distinct eutectic compositions for each solute. The eutectics were prepared by weighing appropriate amounts of the two counterparts in glass vessels and heating them until a homogenous solution was obtained.

The samples of sulfonamides in DESs were prepared by adding an excess amount of the solute to the test tube, followed by an addition of the specific eutectic. The saturated solutions obtained in this manner were placed in an Orbital Shaker Incubator ES-20/60 from Biosan (Riga, Latvia) and heated at 25 °C, 30 °C, 35 °C, or 40 °C for 24 h with stirring at 60 rev/min. Before the measurements, the samples were filtered using a 0.22 µm pore-size PTFE syringe filter. The spectra of the filtered solutions were recorded after the dilution with methanol in the 200 nm–500 nm wavelength range with a 1 nm resolution. No solvatochromic effect was observed in the studied samples, and the analytical wavelength did not shift throughout the experiments. The absorbance values found at the characteristic wavelengths were used together with the linear regression equations to calculate the concentration of the considered solutes. Simultaneously, for the computation of the mole fraction solubility, the density of the samples was determined by weighing 1 mL of the sample on a RADWAG (Radom, Poland) AS 110 R2.PLUS analytical balance with 0.1 mg precision. Three separate samples were measured for each system, and the values were averaged.

### 3.3. Solubility Dataset

The entire solubility dataset used for machine learning, comprising N = 8014 data points, includes the following fifteen active pharmaceutical ingredients: caffeine (CAF), theobromine (THB), theophylline (THP), ferulic acid (FA), edaravone (EDA), ibuprofen (IB), ketoprofen (KP), curcumin (CUR), dapsone (DAP), probenecid (PC), sulfacetamide (SCM), sulfamethazine (SMZ), sulfamethoxazole (SMA), sulfanilamide (SNM), and sulfasalazine (SSZ). The dataset is divided into three subclasses, starting with the deep eutectic solvents (DESs), which include two hydrogen bond acceptors (HBAs): choline chloride (ChCl) (N = 1340) and betaine (BI) (N = 278). Additionally, the newly measured data (N = 128) were included for sulfasalazine (SSZ), sulfamerazine (SMA), sulfamethazine (SMZ), and probenecid (PC). To ensure a high degree of molecular diversity, we also collected data from the literature for each of the included APIs in the neat solvents (N = 2064) and binary solvent mixtures (N = 4332), when available. All data, along with references, are provided in the supporting information (see the MS Excel spreadsheet “SI.xlsx”). We believe this dataset represents the most comprehensive solubility data available for the considered APIs, given the current state of knowledge. Indeed, the dataset covers a wide range of solvent types, including highly polar protic and aprotic solvents, such as water and alcohols on one side and acetone, DMSO, and DMF on the other. The collection also includes representatives of other solvent classes, such as esters and non-polar hydrocarbons. Furthermore, halogenated solvents like dichloromethane, chloroform, and carbon tetrachloride are also present. In total, the solubility data for the 46 different solvents were included for the APIs under consideration. Interestingly, only about 33% of all the possible solute–solvent combinations are available. When accounting for temperature variations, this percentage decreases further. A coverage map, listing the measured and unavailable combinations, is provided in the Supplementary Materials (see Table S2.1). A complete documentation, along with references and solubility values, is included in the supporting materials (see the “Table.S1_neat” spreadsheet in the “SI.xlsx” MS Excel workbook). The subset of data characterizing the binary solvent mixtures is more homogeneous, covering mixtures of water, alcohols, and some other solvents, which are listed in the Supplementary Materials (see Table S2.2). Full documentation, references, and solubility values are also included (see the “Table.S2_bin” spreadsheet in the “SI.xlsx” workbook). Excluding the temperature and concentration dependencies, only about 15% of the possible binary mixtures were studied for the APIs considered in this study. The third subset of the dataset includes the DESs based on choline chloride and betaine as the HBAs, mixed in various proportions with a range of HBDs. The list is available in the Supplementary Materials (see Table S2.3), and further details are available in the “SI.xlsx” file (see “Table.S3_DES” spreadsheet). These DES systems were optimized to tailor the solubility of specific APIs. It is important to note that the addition of water to DESs is known to enhance solubility at moderate concentrations due to the nanostructuring of the HBA; however, this aspect has not been extensively studied for many API-DES systems. Formally, the coverage of possible API-HBA-HBD combinations in our dataset is about 44%, but the inclusion of varying DES component ratios and the added water significantly reduce the actual coverage. To fill the gaps in the API-DES systems, the solubility of four sulfonamides was measured in four choline chloride and betaine DESs, adding N = 128 new data points to the solubility dataset.

### 3.4. COSMO-RS Solubility Computations

Solubility is routinely computed using a combination of first principles and statistical thermodynamics termed COSMO-RS (Conductor-like Screening Model for Real Solvents) [90,91,92]. This two-step approach leverages the macroscopic properties derived from microscopic atomistic modeling, typically employing the density functional theory (DFT) for a detailed structural diversity quantification at the molecular level. The methodology is well established [93,94,95], so only a brief overview is provided here. The initial step involves the generation of the relevant conformations for both the solutes and solvents. A standard protocol for an extensive conformational analysis was applied using the COSMOconf [96]. In the second step, the COSMOtherm package [97] was utilized to determine the physicochemical properties, including the solubility, intermolecular interaction contributions, and sigma potential distributions. This procedure was applied in our previous studies [62,68,69]. Each molecule was represented by up to ten low-energy conformations identified through independent conformational searches in both the gas and condensed phases. The latter is crucial for accounting for the influence of the surrounding environment within the conductor-like screening model. The outcome of this process is a set of “cosmo” and “energy” files, compatible with the latest parameter set (BP_TZVPD_FINE_24.ctd). This corresponds to a two-step computational procedure: first, optimizing the molecular geometries of the most probable conformations using RI-DFT with the B88-VWN-P86 functional and def-TZVP basis set, followed by single-point energy calculations using def2-TZVPD, as implemented in Turbomole Version 7.8 (compiled 23 October 2023) [98].

The estimated solubility values correspond to the solution of the solid–liquid equilibrium under saturated conditions. This can only be achieved if the fusion data are provided as an additional input, which is essential for characterizing the activity of the pure solute under the conditions corresponding to the solubility measurements. From a general thermodynamic perspective, solubility can be computed based on the melting temperature (T_m_), the heat of fusion ($\Delta H_{fus})$, and the heat capacity change upon melting (Δ*C_p_*), using the following equation:

(1) $$\ln\left(a^{s}\right)=-\frac{\Delta G_{\mathrm{fus}}}{\mathrm{RT}}=\frac{\Delta H_{\mathrm{fus}}}{R}\cdot \left(\frac{1}{T_{m}}-\frac{1}{T}\right)-\frac{1}{\mathrm{RT}}\int_{T_{m}}^{T}\Delta C_{p}\mathrm{dT}+\frac{1}{\mathrm{RT}}\int_{T_{m}}^{T}\Delta C_{p}\mathrm{dT}$$

where R is the gas constant and T is the temperature at which the solubility is measured. For many compounds, including all fifteen APIs considered in this study, the experimental values for the melting point and heat of fusion are available [99,100]. However, the data on ∆C_p_ are more difficult to measure and are unavailable for the majority of the solutes. To address this, several simplifications have been proposed [83,101,102,103,104]. In this study, we assume Δ*C_p_* ≈ Δ*S_fus_* ≈ Δ*H_fus_*·*T_m_*^−1^. To ensure the reproducibility of the solubility calculations, all ∆G_fus_ values have been included in the supporting materials, completing the experimental dataset (see MS Excel workbook “SI.xlsx”). The solubility values were determined using the COMSOtherm by solving the full SLE problem. While this method is more computationally intensive than the iterative approaches, it is necessary, particularly for high solubility cases where the iterative procedure fails and inaccurately predicts the total miscibility between the solute and solvent. In contrast, the full SLE approach yields definite solubility values for every case in the dataset.

### 3.5. Molecular Descriptors

**Figure 7:**
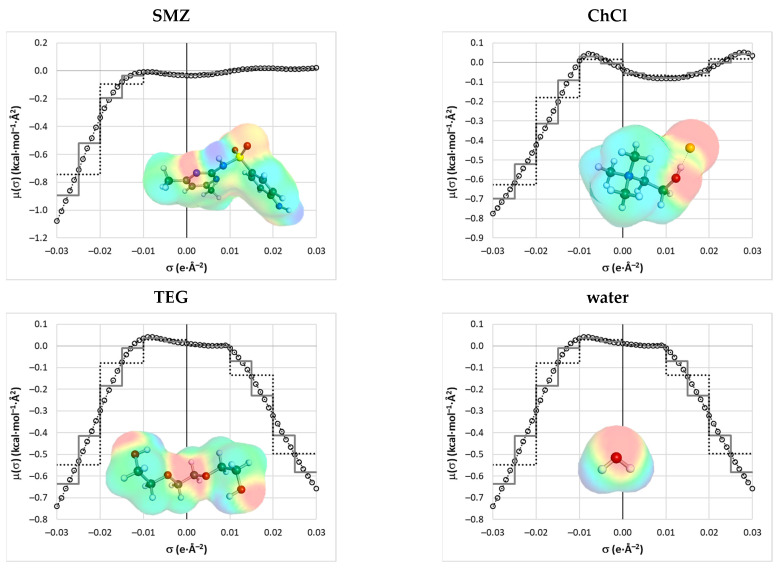
The presentation of the individual σ–potentials enabling the computation of the actual values of Δσ_pot_ for SMZ + ChCl + TEG + water at T = 25 °C. Three types of resolutions are plotted, illustrating the three sets of molecular descriptors used in the machine learning protocol, namely open circles, which denote a full distribution of 61 points (descriptors set C) and two ways of averaging. The grey solid step line represents every six points (descriptor set B), and the dotted black step line characterizes the average over twelve points (descriptor set A).

The selection of molecular descriptors is crucial for the quality of the machine learning models. The descriptors must summarize sufficient information to accurately represent the property being predicted. In the case of solubility modeling, three major requirements are essential for developing a successful model. First, the descriptors must be computable independent of the experimental data for any solute and solvent. Second, they must capture structural diversity, including isomers, rotamers, stereoisomers, and other molecular variations. Lastly, temperature dependence must be accounted for, as it plays a significant role in the equilibrium of saturated systems. These criteria exclude many popular sources of molecular descriptors, such as DRAGON [105] and PADEL [106], which rely either on SMILES strings or on 3D structural data limited to a single conformer. Fortunately, the COSMO-RS offers a comprehensive and effective way to meet these requirements. In this study, a straightforward set of descriptors was applied, leveraging the distribution of σ-potentials. This approach was successfully used in our previous projects [62,63], highlighting the predictive potential of this property derived from charge density distributions. For each solute and solvent, the σ-potential profiles were calculated for their pure, single-component state at a given temperature. The molecular descriptors used for machine learning were computed as the difference between the σ-potential of the pure solute and the σ-potential of the solvent at that specific temperature. For multicomponent solvents, the σ-potential was represented as a weighted value based on the solute-free mole fraction and then used to determine the relative σ-potential profile. Typically, the COSMOtherm program generates σ-potential profiles consisting of 61 points for the σ values ranging from −0.03 to +0.03 e/Å^2^, with a step size of 0.001. Example distributions are provided in Figure 7.

The lines with open circles represent the whole set of 61 points, defining the complete σ-potential of each individual component. In Figure 7, there is also a step function averaging the six subsequent values of the σ potentials provided. It is worth reminding that the entire range of charge densities is often split into three sub-intervals interpreted according to the encoded information. Indeed, the region of σ ∈ [−0.01, +0.01] is typically attributed to hydrophobicity (HYD), the hydrogen bond donicity (HBD) if defined by σ ∈ [+0.01, +0.03], and the hydrogen acceptability (HBA) is addressed to σ ∈ [−0.03, −0.01]. This straightforward interpretation allows for the qualitative and quantitative characteristics of the nature of a given compound to be characterized. For example, water, being an amphiprotic agent, has non-zero contributions for the HBD and HBA regions and is insignificant for the non-polar range. Choline chloride has significantly more hydrophobic contributions compared to the other compounds presented in Figure 7, and due to the large negative volume of chloride anion exposed outwardly, it has a significant HBA range. The step function mimics the whole σ potential distribution with a reduced number of values. This defines the two sets of molecular descriptors, either as a 61-point full record or a simplified 12-point representation. The values presented in Figure 1 were used for the determination of the final sets of descriptors used for the machine learning protocol. For this purpose, the relative potential was defined as follows:

(2) $$\Delta \sigma_{pot}\left(T\right)=\mu_{API}\left(T\right)-\overset{n}{\underset{i}{\sum }}x_{i}^{*}\cdot \mu_{i}\left(T\right)$$

where i iterates over all n components in the solvent mixture and is equal to 1, 2, 3, or 4 for the neat solvents, binary solvent mixtures, dry DESs, and wet DESs, respectively. Hence, the database of σ potentials for every compound at each temperature was prepared and used for the $\Delta \sigma_{pot}\left(T\right)$ computations for every system in the solubility dataset.

Finally, taking advantage of the solubility computations, the energetic contributions of every component were extracted from the output files for characterizing the relative values of Δμ^mix^, ΔE^tot^, ΔE^HB^, ΔE^misfit^, and ΔE^vDW^. The first quantity represents the chemical potential of the mixture, and the rest characterize the contributions of the energetic components, including the relative values of the total energy, hydrogen bonding, the electrostatic contributions, and the non-polar interactions, respectively. These values were computed analogously to the formula provided in Equation (2). The training of non-linear regressors was performed independently for the four sets of descriptors, as defined in Table 2.

The three sets of descriptors A, B, and C differ significantly by the number of values used for training purposes. The comparison of model performances allows for the learning of how detailed a description of the σ potential should be. In the most extended case, i.e., set C, a very detailed representation is taken into account which might lead to overfitting. On the other hand, the representation of the shape of the σ potential by the step function might be effective since the function is generally quite smooth, and the representation of the three major regions just by the four (set B) or two (set A) averaged values might be sufficiently effective as it provides essentially the same information. It is also important to note that the motivation for excluding the values of the COSMO-RS-derived solubility is the relatively low accuracy of these estimates. This is discussed in more detail in the Section 2.2.

### 3.6. Machine Learning Protocol

The machine learning approach employed in this study follows the framework previously established in our earlier research endeavors [62,68,107]. Since the detailed methodology was thoroughly described in earlier publications, only a brief summary is presented here. The solubility prediction model was constructed using the custom Python version 3.10 [108] scripts developed for hyperparameter tuning across 36 distinct regression models. These models span a diverse array of algorithms, including linear regressors, boosting techniques, ensemble methods, nearest neighbors, neural networks, and other types of regressors. Hyperparameter tuning was performed using the Optuna version 3.2 [109], a widely used open-source Python package. The optimization process consisted of 5000 minimization trials, utilizing the tree-structured Parzen estimator (TPE) as the algorithm for sampling the hyperparameter space. To evaluate the performance of each regression model, a custom scoring function was developed, integrating multiple metrics to assess both accuracy and generalizability, as detailed in a previous study [63]. This scoring function incorporates the penalties derived from the learning curve analysis (LCA), performed using the scikit-learn library (version 1.2.2) during the parameter tuning process. It is important to highlight that the custom loss function employed for the solubility prediction models combines several evaluation metrics to ensure both accuracy and robustness, while also addressing the issue of overfitting. A key aspect of the scoring function is the integration of an LCA, which evaluates model performance across different training set sizes. By calculating the mean absolute error (MAE) through a five-fold cross-validation, the function tracks both the training and testing errors at incremental training sizes. Due to the computational demands of an LCA, a limited representation of the learning curve was adopted, using the five points between 50% and 100% of the total dataset. In comparison, our previous work employed only two points (50% and 100%). The extension of an LCA to include more data points aims to provide a more robust early indication of overfitting, albeit at the expense of an increased computational time. The custom loss function also incorporates the mean squared error (MSE) between the predicted and true solubility values, estimated for the training set, along with the penalty terms to further ensure prediction quality. These penalties address the false positive predictions (since the solubility, expressed in a logarithmic form, is expected to be negative) by penalizing any positive predictions based on their frequency. Additionally, the predictions with errors exceeding three standard deviations are classified as outliers and penalized accordingly, encouraging the model to minimize extreme deviations.

From a technical point of view, the whole set of descriptors was split into training (80%) and test (20%) subsets. The evaluation of the models’ performance and their selection was performed using the unseen data by computing the root-mean-square deviations (RMSD) and mean average percentage error (MAPE), which are defined according to the following well-known formula:

(3) $$RMSD=\sqrt{\overset{n}{\underset{i}{\sum }}{\left(\log\left(x_{i}^{exp}\right)-\log\left(x_{i}^{exp}\right)\right)}^{2}/n\;}$$

(4) $$MAPE=\frac{100\%}{n}\overset{n}{\underset{i}{\sum }}\left|\frac{\log\left(x_{i}^{exp}\right)-\log\left(x_{i}^{exp}\right)}{\log\left(x_{i}^{exp}\right)}\right|$$

In addition, the percentage of outliers (%N_out_) representing the percentage of the population with deviations higher than three times the standard deviation was used as the predictive coherence metric.

## 4. Conclusions

The pharmaceutical industry faces various challenges. One of them is the need for a limitation on the energy used, and waste generated during experiments, according to the “green chemistry” framework. Another one is the constant search for new solubilizing media, which plays a pivotal role in the development of new drugs and improving the formulation of already developed drugs. These requirements can be met using the designed deep eutectic solvents. These solvent systems have found widespread use in the pharmaceutical realm; however, the number of potential DES compositions prohibits the experimental testing of all the combinations. The current study addresses this issue by means of a predictive model based on a machine learning protocol, which can limit the experiments required for selecting the optimal eutectic solvents.

The available solubility data for fifteen active pharmaceutical ingredients, namely caffeine, theobromine, theophylline, ferulic acid, edaravone, ibuprofen, ketoprofen, curcumin, dapsone, probenecid, sulfacetamide, sulfamethazine, sulfamethoxazole, sulfanilamide, and sulfasalazine, were the basis for creating the predictive model. A comprehensive exploration of these data led to as many as 8014 data points for these APIs, which comprise the solubility of neat solvents (N = 2064), binary solvent mixtures (N = 4332), and DESs (N = 1618). In this set, there are 128 new data points for probenecid, sulfamethazine, sulfamethoxazole, and sulfasalazine in the various DESs containing choline chloride or betaine, obtained specifically for this study. The molecular predictors used for the purpose of the machine learning process were computed using the COSMO-RS framework. The solubility computations utilizing this method often lead to unsatisfactory results, especially in the case of eutectics, which prohibit the direct use of such values. However, the COSMO-RS can still provide meaningful and useful values in the form of σ-potentials.

In this study, we demonstrated that machine learning can effectively be used for predicting the solubility of new systems and also for filling the gap of lacking solubility data in partially characterized solvent hyperspace. The developed model can be used for an accurate prediction of solubility in untested systems, thereby guiding experimental efforts and optimizing resource allocation. Our extensive analysis revealed that expanding the dataset and exploring diverse solvent combinations significantly enhances the model’s predictive capability. The nu-Support Vector Regressor (nuSVR) has been found to be the most reliable model, achieving high accuracy and generalization, and the most suited for the aim of this paper. Importantly, the choice of descriptor set was found to impact predictive performance more than the inclusion of the COSMO-RS solubility data, suggesting that the models can be streamlined to improve efficiency. It is recommended to use a simplified version of the relative σ-potential in the form of a twelve-step function and omit the solubility values computed in the COSMO-RS. The final robust model is defined by the following parameters of the nuSVR:

- C (6.8251) controls the trade-off between maximizing the margin and minimizing the training error. A higher value of C results in a model that prioritizes fitting the training data closely, potentially at the risk of overfitting.
- Degree (8) is relevant when using polynomial kernels and determines the degree of the polynomial. A degree of eight indicates a highly flexible model capable of capturing the complex relationships in the data.
- Gamma (0.8358) defines the influence of a single training example. A higher gamma value means the model will try to fit the data more closely, as each point has a significant influence on the shape of the decision boundary.
- Max_iter (61,378,442) sets the maximum number of iterations for the algorithm to converge. A high value ensures that the algorithm has sufficient iterations to find an optimal solution, which is especially important for complex models.
- Nu (0.4754) controls the proportion of support vectors and the margin of error, offering a balance between the number of support vectors used and the tolerance for deviations. An optimal value indicates a balanced trade-off, allowing the model to capture the underlying data patterns while controlling the margin of tolerance.

In this configuration, the model is optimized for capturing complex, non-linear relationships in the dataset while maintaining the robustness and generalization capabilities of the extended set of solubility data for DES and non-DES solvents.

## Figures and Tables

**Table 1:** Details of the calibration curves prepared for the sulfonamides used in the solubility dataset extension.

Compound	λ_max_ [nm]	Linear Regression Equation	R^2^	LOD [mg/mL]	LOQ [mg/mL]
probenecid (PC)	246	A = 27.628 × C + 0.001	0.996	0.00126	0.00378
sulfamethazine (SMZ)	269	A = 80.729 × C + 0.002	0.999	0.00052	0.00157
sulfamethoxazole (SMA)	270	A = 69.820 × C + 0.001	0.998	0.00067	0.00202
sulfasalazine (SSZ)	364	A = 87.917 × C + 0.002	0.998	0.00042	0.00127

**Table 2:** Definition of the sets of descriptors used for the hyperparameter tuning of the considered regressors.

Model	Descriptors Set	N_descr_
A1	Δσ- relative potential profiles simplified by step function (N_descr_ = 6) intermolecular interactions (N_descr_ = 5) COSMO-RS derived solubility(N_descr_ = 1)	12
A2	similar to model A1 but without COSMO-RS derived solubility	11
B1	Δσ- relative potential profiles simplified by step function (N_descr_ = 12) intermolecular interactions (N_descr_ = 5) COSMO-RS derived solubility(N_descr_ = 1)	18
B2	similar to model A1 but without COSMO-RS derived solubility	17
C1	Δσ- relative potential profiles as full profile (N_descr_ = 61) COSMO-RS derived solubility	62
C2	as model B1 is without COSMO-RS derived solubility	61

## Data Availability

All data supporting the reported results are available on request from the corresponding author.

## Supplementary Materials

The following supporting information can be downloaded at: https://www.mdpi.com/article/10.3390/molecules29204894/s1. The MS Word document contains the following: Table S1: The mole fraction solubility (∙10^4^) of the sulfonamides in the DESs studied in this work. The HBA:HBD molar ratio is set to 1:2. The standard deviation values are given in parentheses. Table S2.1: the solubility of 15 APIs in the neat solvents. Table S2.2: the solubility of 15 APIs in the binary solvent mixtures. Table S2.3: The solubility of 15 APIs in the DES. The SI.xlsx MS Excel file with the following spreadsheets: Table.S1_neat, Table.S2_bin, and Table.S3_DES. The MS Excel includes the solubility data with references and molecular descriptors used for model development.
