# Supplementary material for: Exploration of the Solubility Hyperspace of Selected Active Pharmaceutical Ingredients in Choline- and Betaine-Based Deep Eutectic Solvents: Machine Learning Modeling and Experimental Validation
Source: Molecules. 2024 Oct 16;29(20):4894. doi: 10.3390/molecules29204894 (PMC11510433; doi:10.3390/molecules29204894)
Supplement: Supplementary file 1 [file molecules-29-04894-s001.zip › SI.pdf]

Supplementary Materials

Exploration of the Solubility Hyperspace of Selected Active Pharmaceutical Ingredients in Choline- and Betaine-Based Deep Eutectic Solvents: Machine Learning Modeling and Experimental Validation

Piotr Cysewski *, Tomasz Jeliński and Maciej Przybyłek

Department of Physical Chemistry, Pharmacy Faculty, Collegium Medicum of Bydgoszcz, Nicolaus Copernicus University in Toruń, Kurpińskiego 5, 85-096 Bydgoszcz, Poland; tomasz.jelinski@cm.umk.pl (T.J.); m.przybylek@cm.umk.pl (M.P.)

* Correspondence: piotr.cysewski@cm.umk.pl

S1. Detailed results of new solubility measurements

Table S1. Mole fraction solubility ($\cdot 10^4$) of sulfonamides in DESs studied in this work. The HBA:HBD molar ratio is set to 1:2. Standard deviation values are given in parentheses.

		**probenecid**		
**HBA:HBD**	**25°C**	**30°C**	**35°C**	**40°C**
ChCl-P2D	8.30(±0.02)	8.54(±0.14)	8.76(±0.03)	9.27(±0.07)
ChCl-ETG	9.47(±0.04)	9.75(±0.08)	10.02(±0.07)	10.57(±0.07)
ChCl-DEG	11.38(±0.08)	11.51(±0.05)	11.79(±0.14)	12.16(±0.09)
ChCl-TEG	12.42(±0.02)	12.58(±0.01)	13.22(±0.11)	13.53(±0.12)
BI-P2D	7.22(±0.10)	7.86(±0.02)	8.24(±0.12)	8.70(±0.11)
BI-ETG	8.33(±0.03)	8.67(±0.03)	9.22(±0.03)	9.61(±0.08)
BI-DEG	10.44(±0.12)	10.95(±0.17)	11.30(±0.10)	11.59(±0.01)
BI-TEG	11.07(±0.01)	11.67(±0.11)	11.77(±0.02)	12.26(±0.02)
		**sulfamethazine**		
**HBA:HBD**	**25°C**	**30°C**	**35°C**	**40°C**
ChCl-P2D	87.27(±0.09)	91.01(±0.01)	94.33(±0.05)	98.41(±0.06)
ChCl-ETG	95.24(±0.07)	98.04(±0.06)	102.27(±0.04)	106.76(±0.04)
ChCl-DEG	111.46(±0.05)	114.31(±0.04)	116.70(±0.06)	119.54(±0.08)
ChCl-TEG	122.50(±0.02)	124.38(±0.04)	127.75(±0.01)	131.50(±0.09)
BI-P2D	75.17(±0.015)	82.16(±0.07)	87.07(±0.07)	90.50(±0.06)
BI-ETG	83.49(±0.09)	86.86(±0.14)	92.20(±0.14)	98.67(±0.04)
BI-DEG	101.09(±0.09)	105.61(±0.03)	107.06(±0.09)	111.56(±0.08)
BI-TEG	104.84(±0.03)	110.17(±0.09)	114.17(±0.11)	118.22(±0.12)
		**sulfamethoxazole**		
**HBA:HBD**	**25°C**	**30°C**	**35°C**	**40°C**
ChCl-P2D	114.62(±0.05)	117.54(±0.07)	123.24(±0.06)	127.88(±0.09)
ChCl-ETG	131.89(±0.07)	137.36(±0.09)	141.71(±0.05)	144.10(±0.08)
ChCl-DEG	161.71(±0.09)	164.17(±0.13)	170.89(±0.04)	175.14(±0.04)
ChCl-TEG	168.09(±0.03)	172.35(±0.02)	178.93(±0.10)	184.38(±0.02)
BI-P2D	100.15(±0.04)	104.44(±0.01)	110.73(±0.03)	117.40(±0.05)
BI-ETG	112.23(±0.05)	119.45(±0.03)	124.99(±0.02)	129.16(±0.04)
BI-DEG	140.27(±0.06)	147.30(±0.15)	155.79(±0.15)	161.11(±0.03)
BI-TEG	145.38(±0.04)	153.48(±0.05)	160.80(±0.03)	167.06(±0.01)
		**sulfasalazine**		
**HBA:HBD**	**25°C**	**30°C**	**35°C**	**40°C**
ChCl-P2D	0.50(±0.08)	0.53(±0.03)	0.56(±0.07)	0.59(±0.01)
ChCl-ETG	0.62(±0.09)	0.65(±0.01)	0.69(±0.06)	0.72(±0.08)
ChCl-DEG	0.71(±0.09)	0.75(±0.07)	0.80(±0.14)	0.83(±0.02)
ChCl-TEG	0.75(±0.05)	0.78(±0.07)	0.82(±0.01)	0.86(±0.02)

BI-P2D	0.41(±0.06)	0.46(±0.10)	0.49(±0.08)	0.52(±0.04)
BI-ETG	0.55(±0.07)	0.58(±0.02)	0.62(±0.06)	0.67(±0.08)
BI-DEG	0.67(±0.09)	0.70(±0.08)	0.75(±0.06)	0.79(±0.05)
BI-TEG	0.69(±0.04)	0.72(±0.02)	0.77(±0.09)	0.81(±0.07)

S2. Solubility dataset

The solubility dataset comprises values retrieved from the literature as well as the results of new measurements done for the purpose of this study. The following fifteen APIs were included: caffeine (CAF), theobromine (THB), theophylline (THP), ferulic acid (FA), edaravone (EDA), ibuprofen (IB), ketoprofen (KP), curcumin (CUR), dapsone (DAP), probenecid (PC), sulfacetamide (SCM), sulfamethazine (SMZ), sulfamethoxazole (SMA), sulfanilamide (SNM), sulfasalazine (SSZ).

Table S2.1. Solubility of 15 APIs in neat solvents.

Illustration of the available solubility values for the studied APIs in neat solvents. The detailed values and references are provided in "Table.S1_neat" spreadsheet of the "SI.xlsx" MS Excel workbook. There were studied 230 saturated systems of 690 possible solute-solvent combinations. The coverage is about 33%, irrespectively of the temperature diversity.

	caffeine	theobromine	theophylline	ferulic acid	edaravone	ibuprofen	ketoprofen	curcumin	dapsone	probenecid	sulfacetamide	sulfamethazine	sulfamethoxazole	sulfanilamide	sulfasalazine
solvent	CAF	THB	THP	FA	EDA	IB	KP	CUR	DAP	PC	SCM	SMZ	SMA	SNM	SSZ
water	+	+	+	+	+	+	+	+	+	-	+	+	+	+	+
Methanol	+	+	+	+	+	+	+	+	+	+	-	+	+	+	+
Ethanol	+	+	+	+	+	+	+	+	+	+	+	+	+	+	+
1-propanol	+	+	+	-	+	+	-	+	+	+	+	+	+	+	+
isopropanol	+	-	-	+	+	+	+	+	+	+	-	+	-	+	-
n-Butanol	+	-	-	+	+	+	+	+	+	+	+	+	+	+	-
2-butanol	-	-	-	+	+	-	-	-	+	-	-	+	-	+	-
Isobutanol	-	-	-	-	+	+	-	-	+	+	-	+	-	-	-
3-methyl-1-butanol	-	-	-	-	-	+	-	-	-	-	-	-	-	-	-
1-Pentanol	+	-	-	-	+	+	-	-	+	+	+	+	+	+	-
1-octanol	+	+	+	-	-	+	-	-	-	-	+	+	+	+	+
carbitol	+	-	-	+	-	-	-	-	-	-	-	-	-	-	-
ethylene glycol	+	-	-	+	-	-	-	-	-	-	+	+	+	+	-
propylene glycol	+	-	-	+	-	+	+	+	-	-	+	-	-	+	-
diglyme	-	-	-	-	+	-	-	-	-	-	-	-	-	-	-
Triglyme	-	-	-	-	+	-	-	-	-	-	-	-	-	-	-
Tetraglyme	-	-	-	-	+	-	-	-	-	-	-	-	-	-	-
2,4-dimethylphenol	-	-	-	-	+	-	-	-	-	-	-	-	-	-	-
Methyl acetate	-	-	-	-	-	-	-	-	+	+	-	-	-	-	-
Ethyl propionate	-	-	-	-	-	-	-	-	+	-	-	-	-	-	-
Butyl acetate	-	-	-	-	-	-	-	-	+	+	-	+	-	+	-
Isopropyl acetate	-	-	-	-	-	+	-	-	+	-	-	-	-	-	-
ethyl lactate	+	-	-	+	-	-	-	-	-	-	-	-	-	-	-
Ethyl acetate	+	+	+	+	+	+	+	+	+	+	-	+	-	+	-
isopropyl myristate	-	-	-	-	-	+	-	-	-	-	-	-	-	-	-
methyl tert-butyl ether	-	-	-	-	-	-	-	-	-	+	-	-	-	-	-
acetone	+	+	+	-	+	+	-	+	+	+	-	+	+	+	+
2-butanone	-	-	-	-	-	+	-	-	-	-	-	-	-	-	-
3-methyl-2-butanone	-	-	-	-	-	+	-	-	-	-	-	-	-	-	-
4-methyl-2-pentanone	-	-	-	-	-	+	-	-	-	-	-	-	-	-	-
DMSO	+	-	-	+	+	-	-	+	-	-	+	+	+	+	-
Tetrahydrofuran	-	-	-	-	-	-	-	+	-	-	-	-	-	-	-
N-methyl-2-pyrrolidone	+	-	-	-	+	-	-	-	-	-	-	-	-	-	-
Acetonitrile	+	-	-	-	+	+	+	+	-	-	+	+	+	+	-
DMF	+	-	-	-	-	-	-	-	-	-	+	+	+	+	-
4FM	-	-	-	-	+	-	-	-	-	-	-	-	+	+	-
1,4-dioxane	+	-	-	-	+	-	+	-	+	-	+	+	+	+	-
Dichlormethane	+	-	-	-	+	+	-	-	-	-	-	-	-	-	-
Chloroform	+	-	-	-	-	+	-	-	-	-	-	+	+	+	-
Carbon tetrachloride	+	-	-	-	-	-	-	-	-	-	-	-	-	-	-
n-Hexane	-	-	-	-	-	-	-	+	-	-	-	+	-	+	+
n-Heptane	-	-	-	-	-	+	-	+	-	-	-	-	-	-	-
cyclohexane	-	-	-	-	-	+	+	-	-	-	+	+	+	+	-
benzene	-	-	-	-	-	-	-	-	-	-	-	-	-	+	-
Toluene	-	-	-	-	+	+	+	-	-	-	-	+	-	+	-
p-Xylene	-	-	-	-	-	-	-	+	-	-	-	-	-	-	-

Table S2.2. Solubility of 15 APIs in binary solvents mixtures.

Illustration of the available solubility values for the studied APIs in binary solvent mixtures. The detailed values and references are provided in "Table.S2_bin" spreadsheet of the "SI.xlsx" MS Excel workbook. There were studied 89 types of saturated systems of 585 possible solute-solvent combinations. The coverage is about 15%, irrespectively of the temperature diversity and concentration dependencies.

		caffeine	theobromine	theophylline	ferulic acid	edaravone	ibuprofen	ketoprofen	curcumin	dapsone	probenecid	sulfacetamide	sulfamethazine	sulfamethoxazole	sulfanilamide	sulfasalazine
		CAF	THB	THP	FA	EDA	IB	KP	CUR	DAP	PC	SCM	SMZ	SMA	SNM	SSZ
water	methanol	+	+	+	-	+	+	+	-	+	-	-	+	-	+	-
water	ethanol	-	-	-	-	+	+	-	+	+	-	+	+	-	+	-
water	1-Propanol	-	-	+	-	-	-	-	+	+	-	-	+	-	-	-
water	isopropanol	-	-	-	+	+	-	-	+	+	-	-	+	-	-	-
water	1-butanol	-	-	+	-	-	-	-	-	-	-	-	-	-	-	-
water	ethylene glycol	-	-	-	-	-	-	-	-	-	-	-	+	-	-	-
water	propylene glycol	-	-	-	-	-	+	+	+	-	-	+	+	-	+	-
water	DMSO	+	+	+	-	-	-	-	-	-	-	-	+	+	+	-
water	DMF	+	+	+	-	-	-	-	-	-	-	-	+	+	+	-
water	4-Formylmorpholine	-	-	-	-	-	-	-	-	-	-	-	-	+	+	-
water	1,4-dioxane	+	+	+	-	-	-	-	-	+	-	-	+	+	+	-
water	acetonitrile	+	-	-	-	-	-	-	-	-	-	-	+	-	+	-
water	acetone	+	+	-	-	-	-	-	-	+	-	-	-	-	-	-
methanol	ethanol	-	-	-	-	-	-	-	-	-	-	-	-	-	+	-
methanol	acetonitrile	-	-	-	-	+	-	-	-	-	-	-	+	-	-	-
methanol	ethyl acetate	-	-	-	-	+	-	-	-	-	-	-	-	-	-	-
methanol	toluene	-	-	-	-	-	-	-	-	-	-	-	-	-	+	-
methanol	chloroform	-	-	-	-	-	-	-	-	-	-	-	-	-	+	-
methanol	1-octanol	-	-	-	-	-	-	-	-	-	-	-	+	-	-	-
methanol	carbon tetrachloride	+	-	-	-	-	-	-	-	-	-	-	-	-	-	-
ethanol	acetonitrile	-	-	-	-	+	-	-	-	-	-	-	-	-	-	-
ethanol	N-methyl-2-pyrrolidone	+	-	-	-	-	-	-	-	-	-	-	-	-	-	-
ethanol	Acetone	-	-	-	-	-	-	-	-	-	-	-	-	-	+	-
ethanol	Propylene glycol	-	-	-	-	-	+	-	-	-	-	-	-	-	-	-
ethanol	ethyl acetate	+	-	-	-	+	-	-	-	+	-	-	-	-	-	-
ethanol	toluene	-	-	-	-	-	-	-	-	-	-	-	-	-	+	-
ethanol	carbitol	+	-	-	-	-	-	-	-	-	-	-	-	-	-	-
ethanol	chloroform	-	-	-	-	-	-	-	-	-	-	-	-	-	+	-
n-propanol	acetonitrile	-	-	-	-	+	-	-	-	-	-	-	-	-	-	-
n-propanol	ethyl acetate	-	-	-	-	+	-	-	-	-	-	-	-	-	-	-
1-propanol	N-methyl-2-pyrrolidone	+	-	-	-	-	-	-	-	-	-	-	-	-	-	-
isopropanol	N-methyl-2-pyrrolidone	+	-	-	-	-	-	-	-	-	-	-	-	-	-	-
ethylene glycol	N-methyl-2-pyrrolidone	+	-	-	-	-	-	-	-	-	-	-	-	-	-	-
propylene glycol	N-methyl-2-pyrrolidone	+	-	-	-	-	-	-	-	-	-	-	-	-	-	-
Acetone	Ethyl Acetate	-	-	-	-	-	-	-	-	-	-	-	-	-	+	-
Acetone	Toluene	-	-	-	-	-	-	-	-	-	-	-	-	-	+	-
acetone	tetrahydrofuran	-	-	-	-	-	-	-	+	-	-	-	-	-	-	-
tetrahydrofuran	acetonitrile	-	-	-	-	-	-	-	+	-	-	-	-	-	-	-
tetrahydrofuran	n-hexane	-	-	-	-	-	-	-	+	-	-	-	-	-	-	-

Table S2.3. Solubility of 15 APIs in DES.

Illustration of the available solubility values for the studied APIs in binary solvent mixtures. The detailed values and references are provided in “Table.S3_DES” spreadsheet of the “SI.xlsx” MS Excel workbook. There were studied 118 types of saturated systems of 270 possible solute-solvent combinations. The coverage is about 44%, however, the temperature diversity and the concentration dependencies were not included in this estimate.

		caffeine	theobromine	theophylline	ferulic acid	edaravone	ibuprofen	ketoprofen	curcumin	dapsone	probenecid	sulfacetamide	sulfamethazine	sulfamethoxazole	sulfanilamide	sulfasalazine
**HBA**	**HBD**	CAF	THB	THP	FA	EDA	IB	KP	CUR	DAP	PC	SCM	SMZ	SMA	SNM	SSZ
choline chloride	P2D	-	-	-	+	+	+	+	-	+	-	-	-	-	-	-
choline chloride	DEG	-	-	-	+	+	+	+	-	+	-	-	-	-	-	-
choline chloride	TEG	-	-	-	+	+	+	+	-	+	-	-	-	-	-	-
choline chloride	B3D	-	-	-	+	+	+	+	-	+	-	-	-	-	-	-
choline chloride	GLY	+	+	+	+	+	+	+	+	+	+	+	+	+	+	+
choline chloride	ETG	-	-	-	+	+	+	+	-	+	-	-	-	-	-	-
choline chloride	FRU	+	+	+	-	-	-	-	+	-	+	+	+	+	+	+
choline chloride	GLU	+	+	+	-	-	-	-	+	-	+	+	+	+	+	+
choline chloride	SOR	+	+	+	-	-	-	-	+	-	+	+	+	+	+	+
choline chloride	XYL	+	+	+	-	-	-	-	+	-	+	+	+	+	+	+
choline chloride	SUC	+	+	+	-	-	-	-	+	-	+	+	+	+	+	+
choline chloride	MAL	+	+	+	-	-	-	-	+	-	+	+	+	+	+	+
betaine	P2D	-	-	-	+	-	+	+	-	-	-	-	-	-	-	-
betaine	DEG	-	-	-	+	-	+	+	-	-	-	-	-	-	-	-
betaine	TEG	-	-	-	+	-	+	+	-	-	-	-	-	-	-	-
betaine	B3D	-	-	-	+	-	+	+	-	-	-	-	-	-	-	-
betaine	GLY	-	-	-	+	-	+	+	-	-	-	-	-	-	-	-
betaine	ETG	-	-	-	+	-	+	+	-	-	-	-	-	-	-	-
